# Integrated analysis of m^6^A mRNA methylation in rats with monocrotaline-induced pulmonary arterial hypertension

**DOI:** 10.18632/aging.203230

**Published:** 2021-07-26

**Authors:** Yunhong Zeng, Ting Huang, Wanyun Zuo, Dan Wang, Yonghui Xie, Xun Wang, Zhenghui Xiao, Zhi Chen, Qiming Liu, Na Liu, Yunbin Xiao

**Affiliations:** 1 Academy of Pediatrics, University of South China, Changsha 410007, China; 2 Department of Cardiology, Hunan Children’s Hospital, Changsha 410007, China; 3 Department of Utrasound, Hunan Children’s Hospital, Changsha 410007, China; 4 Department of Cardiovascular Medicine, Second Xiangya Hospital, Central South University, Changsha 410011, China; 5 Department of Intensive Care Unit, Hunan Children’s Hospital, Changsha 410007, China

**Keywords:** pulmonary arterial hypertension, N6-methyladenosine, FTO, YTHDF1

## Abstract

Background: N6-methyladenosine (m^6^A) modification is one of the most common chemical modifications of eukaryotic mRNAs, which play an important role in tumors and cardiovascular disease through regulating mRNA stability, splicing and translation. However, the changes of m^6^A mRNA and m^6^A-related enzymes in pulmonary arterial hypertension (PAH) remain largely unexplored.

Methods: MeRIP-seq was used to identify m^6^A methylation in lung tissues from control and MCT-PAH rats. Western blot and immunofluorescence were used to evaluate expression of m^6^A-related enzymes.

Results: Compared with control group, m^6^A methylation was mainly increased in lung tissues from MCT-PAH rats. The up-methylated coding genes in MCT-PAH rats were primarily enriched in processes associated with inflammation, glycolysis, ECM-receptor interaction and PDGF signal pathway, while genes with down-methylation were enriched in processes associated with TGF-β family receptor members. The expression of FTO and ALKBH5 downregulated, METTL3 and YTHDF1 increased and other methylation modification-related proteins was not significantly changed in MCT-PAH rats lung tissues. Immunofluorescence indicated that expression of FTO decreased and YTHDF1 increased in small pulmonary arteries of MCT-PAH rats.

Conclusion: m^6^A levels and the expression of methylation-related enzymes were altered in PAH rats, in which FTO and YTHDF1 may play a crucial role in m^6^A modification.

## INTRODUCTION

Pulmonary arterial hypertension (PAH) is a progressive disease with unfavorable treatment outcomes and poor prognosis [1]. The pathogenic mechanisms of PAH primarily include inflammation, immune abnormalities, oxidative stress and epigenetic changes, which cause pulmonary vasoconstriction and vascular remodeling [2]. Typical pathological characteristics of PAH are intimal hyperplasia resulting in cavity stenosis or occlusion, muscular thickening in the small pulmonary arterioles, adventitial fibrosis and *in situ* thrombosis, which is related to abnormalities in the proliferation and apoptosis of endothelial cells and pulmonary arterial smooth muscle cells and extracellular matrix remodeling [2, 3]. Currently available drugs aim mainly to decrease pulmonary vasoconstriction rather than reverse vascular remodeling [4]. However, end-stage PAH is caused mainly by vascular remodeling [5, 6], thus, the need to identify a new approach to treat vascular remodeling is extremely urgent.

Numerous studies have indicated that epigenetic modifications, including DNA methylation, histone modifications and microRNA dysregulation, play important roles in regulating PAH [7]. RNA, an intermediate in the flow of genetic information from DNA to proteins, is an important part of the central dogma of molecular biology, and its various chemical modifications mediate the regulation of many biological processes [8]. N6-methyladenosine (m^6^A) modification of RNA transcripts is the most prevalent modification in many classes of RNA [9]. m^6^A modification is a critical regulator of mRNA stability, protein expression, and several other cellular processes [10]. Recently, a transcriptome-wide map of the m^6^A modification of circular RNAs (circRNAs) in hypoxia-mediated pulmonary hypertension (HPH) was constructed, and the level of m^6^A circRNAs was found to be decreased in HPH [11]. However, changes in m^6^A mRNA methylation and the expression levels of m^6^A-related enzymes in PAH lung tissues remain largely unexplored.

m^6^A modification is one of the most abundant and prevalent internal modifications of mRNA, and like DNA methylation, it is dynamically regulated by various m^6^A-related enzymes including writers, erasers and readers [12]. The installation of m^6^A is catalyzed by “writers”, such as the multicomponent methyltransferase complex consisting of Methyltransferase Like 3 and 14 (METTL3, METTL14) [13, 14]. “Erasers”, including fat mass and obesity-associated protein (FTO) and alkB homolog 5 (ALKBH5), are responsible for catalyzing the removal of m^6^A methylation [15, 16]. “Readers”, such as the YT521-B homology (YTH) domain-containing protein family, which includes YTHDF (YTHDF1, YTHDF2, YTHDF3), YTHDC1, and YTHDC2, specifically recognize m^6^A and regulate the splicing, localization, degradation and translation of RNA [17, 18].

In this study, we used methylated RNA immunoprecipitation sequencing (MeRIP-seq) to establish the transcriptome-wide m^6^A methylome profile of lung tissue from rats with monocrotaline (MCT)-induced PAH. Then, western blot and immunofluorescence were used to detect methylation modification-related enzymes. Moreover, we screened potential target transcripts involved in PAH.

## RESULTS

### Hemodynamic test

**Figure 1:**
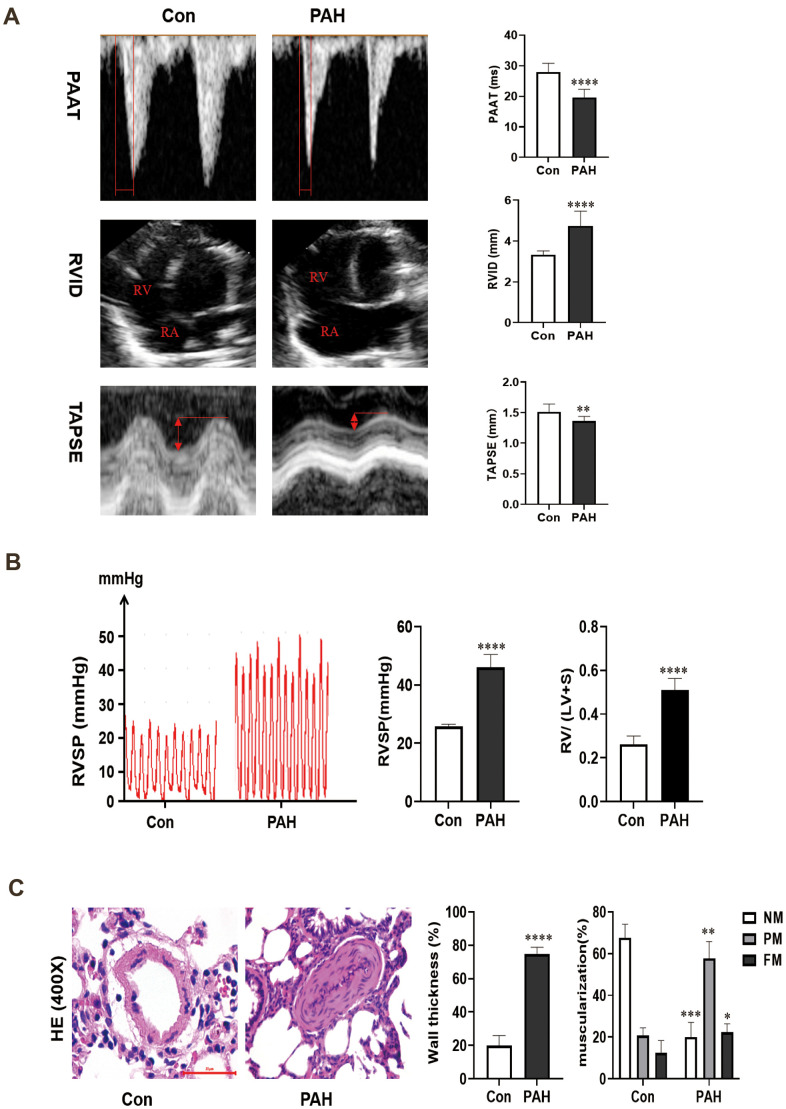
(**A**) Changes of echocardiography in rats after intraperitoneal injection of monocrotaline (MCT) for 4 weeks. (**B**) The hemodynamic test results of the two groups. Compared with the control group, the right ventricular systolic pressure was significantly increased in the PAH group, and RV/ (LV + S) also increased in the PAH group. (**C**) Pulmonary artery HE staining images of the control group and PAH group were obtained under microscopy. Pulmonary artery remodeling was observed in PAH group compared with control group. *P < 0.05, **P < 0.01, ***P < 0.001, ****P < 0.0001.

The pulmonary artery velocity profile in the PAH group was dagger-shaped, and the pulmonary artery blood flow acceleration time (PAAT) was correspondingly reduced compared to that in the control group (19.60 ± 2.54 vs 27.88 ± 2.71 ms, *p* < 0.0001). In addition, the ventricular septum was significantly shifted to the left ventricle, and the right ventricle was enlarged in the PAH group compared to the control group (4.74 ± 0.69 vs 3.34 ± 0.17 mm, *p* < 0.0001). Compared to that in the control group, the tricuspid annular plane systolic excursion (TAPSE) was greatly reduced in the PAH group (1.36 ± 0.07 vs 1.51 ± 0.12 mm, *p* < 0.01) (Figure 1A). Four weeks after MCT injection, the right ventricular systolic pressure (RVSP) in the PAH group was elevated (45.97 ± 4.25 mmHg vs 25.74 ± 0.73 mmHg, *P* < 0.0001), and the RV/ (LV + S) value was increased in the PAH group compared with the control group (0.51± 0.05 vs 0.26 ± 0.03 g, *p* < 0.0001) (Figure 1B).

### Hematoxylin-eosin (HE) staining

The results of HE staining showed that the medial muscle of the pulmonary artery was significantly thickened (74.67 ± 3.94% vs 19.93 ± 5.63%, *P* < 0.0001) and that the vascular lumen was reduced in the PAH group compared with the control group. Moreover, in the control group, 67.67 ± 5.25% of the arterioles were non-muscularized (NM) vessels, and 12.33 ± 4.92% were fully muscularized (FM) vessels. In contrast, partially muscularized vessels (PM) and FM vessels showed a greater proportion (57.67 ± 6.60% and 22.33 ± 2.49%) in MCT-PAH rats, while NM vessels occupied a lower proportion (20.00 ± 5.72%) (Figure 1C). The results of hemodynamic analysis and HE staining indicated that the rat model of PAH had been successfully established.

### Methylation profile of lung tissue from rats with PAH

**Figure 2:**
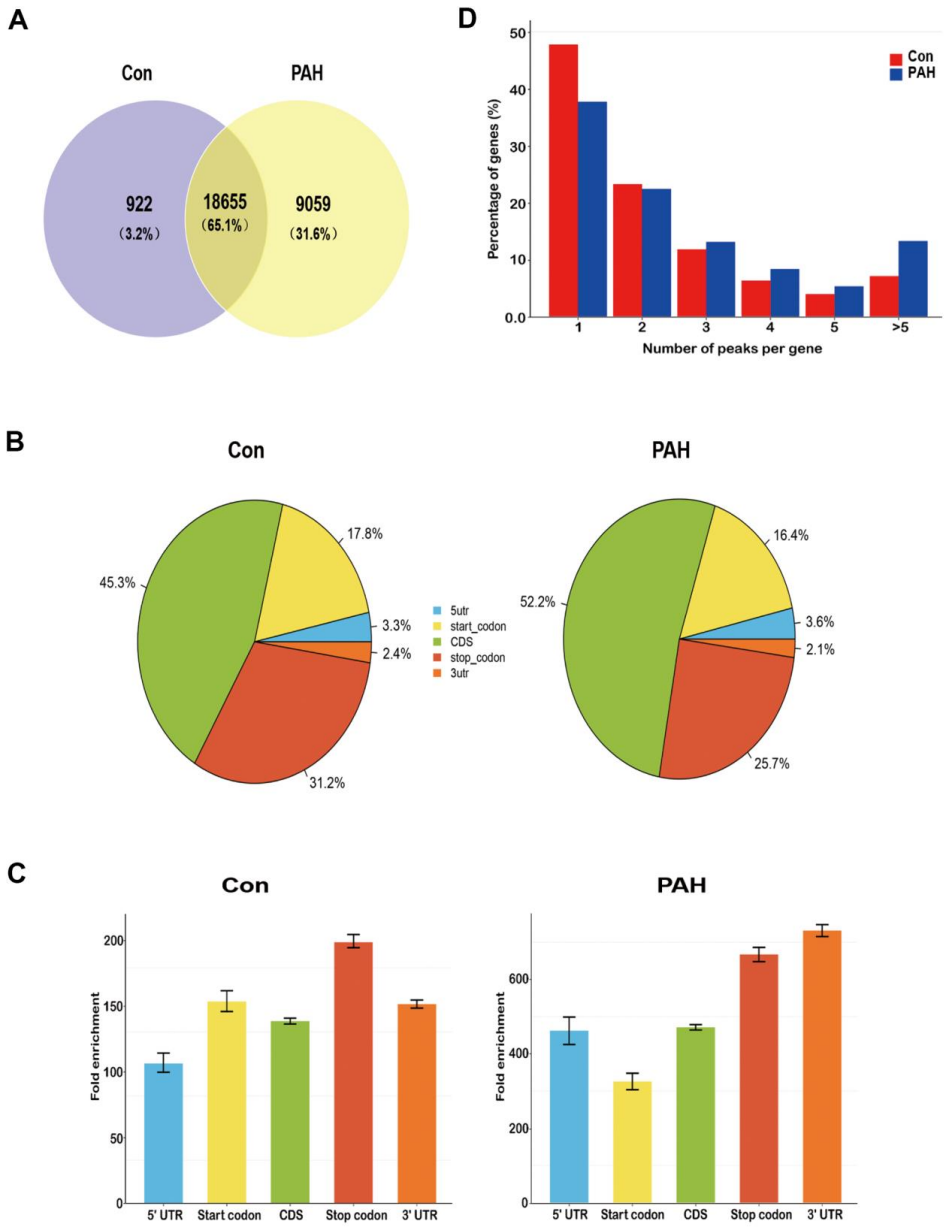
**Overview of N6-methyladenosine methylation within mRNAs in the control and MCT groups.** (**A**) Venn diagram showing the overlap of m^6^A peaks within mRNAs in two groups. (**B**) Pie charts showing the percentage of m^6^A peaks in five non-overlapping segments of transcripts. Both the control group and the PAH group had the most abundant m^6^A peak in the coding sequence. (**C**) Distributions of fold enrichment of m^6^A peaks in five segments. The mean fold enrichment in the stop codon segments was the largest in the control group, while that value in 3' UTR was the largest in the PAH group. Error bars represent the standard error of the mean. (**D**) Proportion of genes harboring different numbers of m^6^A peaks in two groups. Most genes have only one m^6^A peak.

Lung tissues were collected from the two groups. MeRIP-seq analysis identified 922 nonoverlapping m^6^A sites in the control group, 9059 nonoverlapping m^6^A sites in the PAH group, and 18655 overlapping m^6^A sites between the two groups (Figure 2A). Nearly one-third of the m^6^A sites were found exclusively in the PAH group, suggesting that methylation modification plays an important role in PAH.

The enrichment peaks were annotated to the nearest gene by bioinformatic analysis approaches, thus mapping the genome through the use of annotation information. We systematically classified these m^6^A sites into five transcript regions—5’UTRs, 3’UTRs, stop codons, start codons and coding sequences (CDs)—and found that the m^6^A sites were distributed mostly in CDs, stop codons, and start codons in both groups (Figure 2B). Notably, the mean fold enrichment was largest in the stop codon segments in the control group but the 3’UTR regions in the PAH group (Figure 2C), indicating that there are different methylation patterns between normal and disease. Meanwhile, the distribution pattern of methylated modifications was similar to previous studies [19, 20].

Notably, 47.7% of the m^6^A-modified coding genes in the control group and 36.2% of those in the PAH group contained only one m^6^A peak, consistent with a single m^6^A site or a cluster of adjacent m^6^A residues. The next highest percentage contained two m^6^A peaks, while a relatively small percentage contained three or more peaks (Figure 2D), which is agree with the trend of the proportions previously reported for the pig liver [21] and mouse heart [22].

Next, differentially methylated m^6^A sites (DMMSs) between the groups were identified by diffReps with the following default screening criteria: an FDR ≤ 0.0001 and a fold change ≥ 2. We selected 3298 DMMSs between the two groups. A total of 777 m^6^A sites exhibited decreased methylation, and 2521 exhibited increased methylation. On average, 23.6% and 76.4% of the m^6^A sites exhibited prominently decreased and increased methylation, respectively, in PAH lung tissues relative to control lung tissues (Table 1). Tables 2, 3 show the top ten genes with increased and decreased methylation.

**Table 1:** Total numbers of differentially methylated N6-methyladenosine peaks and associated gene.

**Item**	**Hypermethylated peak**	**Hypermethylated gene**	**Hypomethylated peak**	**Hypomethylated gene**
mRNA	2521	1261	777	568

**Table 2:** Top ten up-regulated genes.

**chrom**	**txStart**	**txEnd**	**GeneName**	**Foldchange**
chr10	14521318	14521596	Tpsab1	1643.7
chr2	124777380	124777456	Cpa3	852.7
chr6	39260307	39260379	LOC257642	528.3304473
chr15	38492079	38492215	Cma1	523.6
chr1	235026074	235026209	Ms4a2	454.3
chr10	14519180	14519530	Tpsab1	422.8
chr15	38920068	38920123	Mcpt2	399.3
chr6	147108214	147108505	AABR06046430.3	375.8
chr6	148133714	148134006	Ighg	324.7
chr15	38499889	38500287	Mcpt1l1	317.4916885

**Table 3:** Top ten down-regulated genes.

**chrom**	**txStart**	**txEnd**	**GeneName**	**Foldchange**
chr13	94156781	94157040	Mpz	144.4
chr15	37540300	37540399	Myh7	127.2
chr8	118499941	118500097	Als2cl	111.7
chr1	32944021	32944397	Zfp72	110.5
chr2	230629248	230629515	Mybphl	93.6
chr17	67605795	67605900	Ryr2	89.8
chr17	17414465	17414480	Iars	88.2
chr2	227847959	227848030	Wdr77	87.9
chr5	163485561	16485644	Arhgef19	83.9
chr2	228004667	228004738	Wdr77	82.9

**Figure 3:**
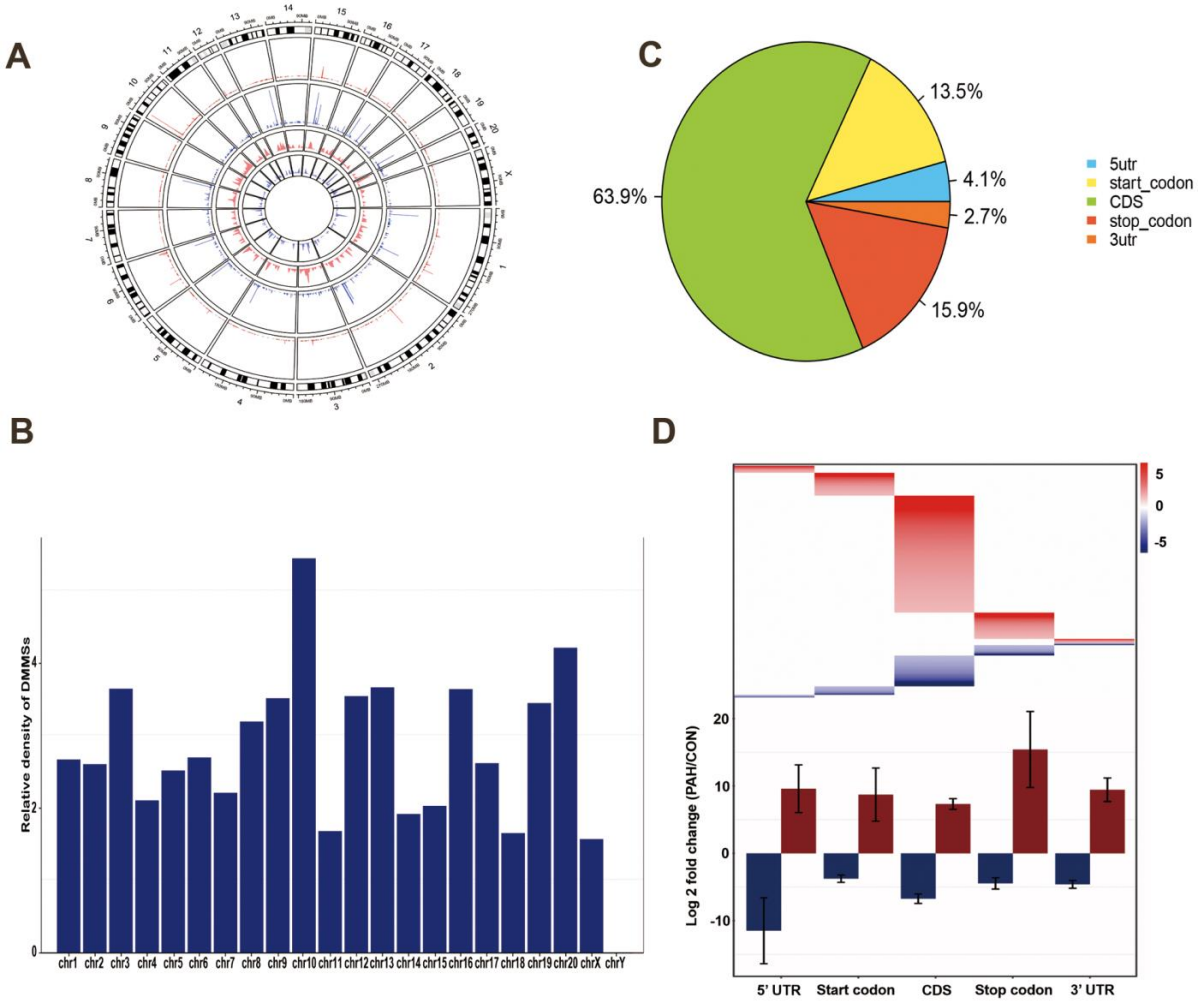
**Distribution of differentially methylated N6-methyladenosine sites.** (**A**) Circos plots was used to plot the chromosomal distribution and genomic density of all DMM sites on genome. In the plot, red corresponds to up-methylated sites (gain of methylation) and blue corresponds to down-methylated sites (loss of methylation). Two histogram tracks were closer to the rat genome showed the chromosomal distribution of up-methylated sites and down-methylated sites, respectively. Two genomic density tracks were father from the rat genome showed the genomic density of up-methylated sites and down-methylated sites, respectively. (**B**) Relative occupancy of differentially methylated m^6^A sites in each chromosome normalized by length of the respective chromosome. (**C**) Pie chart showing the percentage of DMM peaks in five non-overlapping segments. Among them the largest proportion is the coding sequence. (**D**) Statistics of fold change of DMM peaks in five segments. The upper heatmap shows the distribution of the fold change, while the lower histogram shows the mean of the fold change. Error bars represent the standard error of the mean. (DMM: Differentially methylated N6-methyladenosine).

To assess their distribution profiles, all DMMSs within mRNAs were mapped to chromosomes (Figure 3A). The five chromosomes harboring the most DMMSs were chromosomes 1, 2, 4, 3 and 5. However, when the number of DMMSs harbored by a chromosome was normalized by the length of that chromosome, chromosomes 10, 20, 3, 13 and 16 were the five chromosomes with the highest relative densities of DMMSs (Figure 3B). We further investigated the regions with DMMSs in mRNA and found that most were located in CDs (Figure 3C). Moreover, most m^6^A sites in the stop codons with upregulated methylation showed the highest fold change, however, most m^6^A sites in the 5’UTRs with downregulated methylation showed the highest fold change (Figure 3D). Figure 3B, 3D suggesting the preferences of methylation and demethylation genome wide in the PAH group compare to the control group.

### Differentially methylated RNAs are involved in important biological pathways

**Figure 4:**
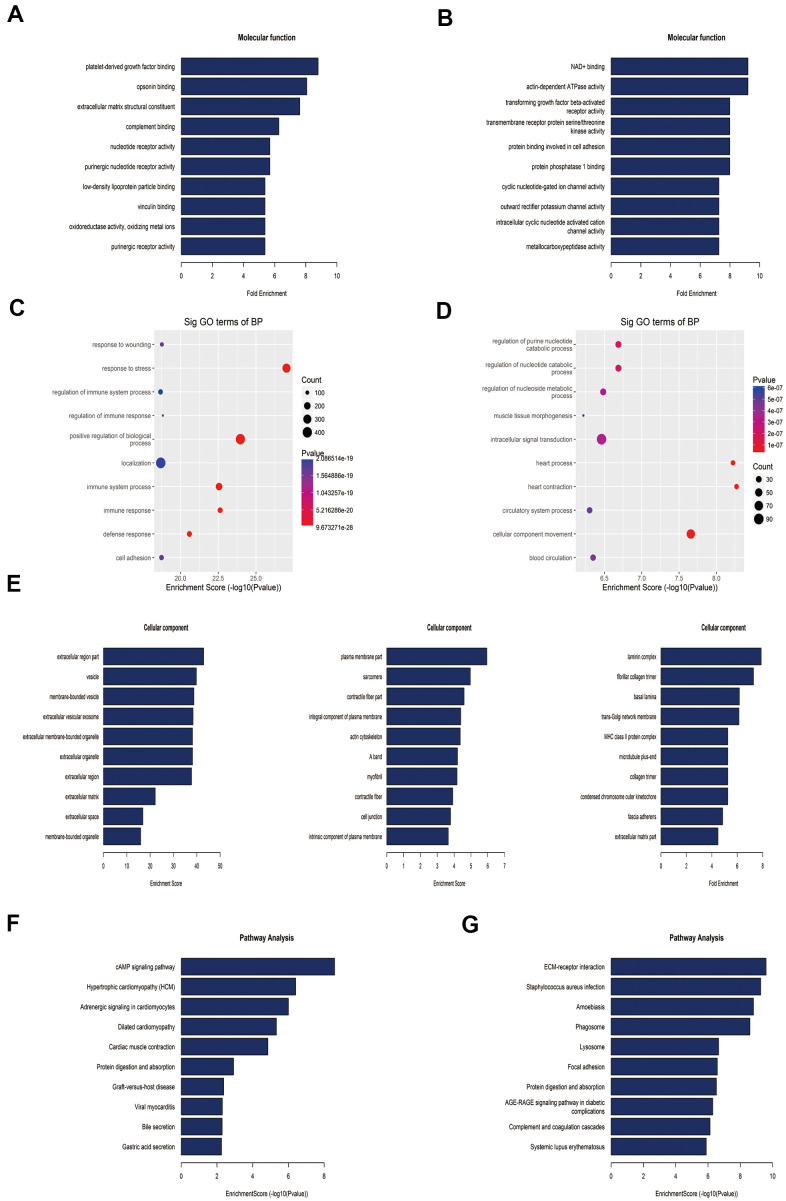
**Gene ontology and Kyoto Encyclopedia of Genes and Genomes analyses of coding genes harboring differentially methylated N6-methyladenosine sites.** (**A**) Bar plot showing the top ten enrichment scores of the molecular function for the up-methylated genes. (**B**) Bar plot showing the top ten enrichment scores of molecular functions of the down-methylated genes. (**C**) The top ten gene ontology terms of biological processes were significantly enriched for the up-methylated genes. (**D**) The top ten gene ontology terms of biological process significantly enriched for down-methylated genes. (**E**) In the cellular component category, the lung tissues of rats mainly contain genes with DMMSs. (**F**) Bar plot showing the top ten enrichment scores of the significant enrichment pathway for the up-methylated genes. (**G**) Bar plot showing the top ten enrichment scores of the significant enrichment pathways of the down-methylated genes.

To reveal the functions of m^6^A in the lung tissues of rats with MCT-induced PAH, protein-coding genes containing DMMSs were selected for GO enrichment and KEGG pathway analyses. In the molecular function (MF) category, genes with up-methylated at m^6^A sites were significantly (*p*< 0.05) enriched in the platelet-derived growth factor (PDGF) binding, opsonin binding, extracellular matrix structural constituent, and complement binding categories (Figure 4A), while genes with down-methylated at m^6^A sites were highly enriched in the NAD^+^ binding, actin-dependent ATPase activity, transforming growth factor beta (TGF-β)-activated receptor activity, and transmembrane receptor protein serine/threonine kinase activity categories, among others (Figure 4B). Notably, studies have shown that the PDGF signaling pathway plays an important role in PAH. In the biological process (BP) category, immune-related processes were most enriched in rat lung tissues with upregulated methylation (Figure 4C), while heart-related processes, cellular component movement and regulation of purine nucleotide catabolic process were enriched in tissues with downregulated methylation (Figure 4D). In the cellular component category, genes containing DMMSs in lung tissues were enriched mainly in the extracellular region, plasma membrane, laminin complex and so on (Figure 4E).

Furthermore, genes with down-methylated m^6^A sites were found to be significantly (*p*< 0.05) involved in cardiovascular disease-related pathways, such as the cAMP signaling pathways, hypertrophic cardiomyopathy, adrenergic signaling in cardiomyocytes, and dilated cardiomyopathy (Figure 4F), while those with up-methylated m^6^A sites were involved in ECM-receptor interaction, staphylococcus aureus infection and so on (Figure 4G).

MeRIP-seq showed that m^6^A of COL1A1/2 were up-methylated, which were associated with PDGF binding and extracellular matrix structural constituent in molecular function by GO analysis (Table 4). KEGG Pathway analysis indicated that COL1A1/2 related to ECM-receptor interaction, PI3K/AKT signaling pathway and Platelet activation (Table 5). Meanwhile, mRNA m^6^A levels of HK3, GPI, LDHA and PKM were up-regulated, which were associated with glycolysis/gluconeogenesis pathway by KEGG Pathway analysis (Table 5). m^6^A of NF-κB was also up-methylated, which was connected with chemokine signaling pathway, T/B cell receptor signaling pathway and HIF-1 signaling pathway (Table 5). More importantly, ACVRL1, ENG, SMAD6 and SMAD9 had down-regulated methylation, which related to TGF-β signal pathway in molecular function, biological process or KEGG pathway (Table 6).

**Table 4:** Molecular function of upregulated m^6^A methylation.

**GO.ID**	**Term**	**Count**	***P*-value**	**Genes**
GO:0005201	extracellular matrix structural constituent	17	7.29608E-12	COL1A1/ COL1A2/ELN/EMILIN1/ LAMC1/FN1/COL12A1/COL4A1/ LAMB1/COL4A4/COL4A2/COL4A3/ PXDN/FBN1/COL3A1/COL5A2/COL5A1
GO:0048407	platelet-derived growth factor binding	6	1.97812E-05	COL1A1/ COL1A2/COL4A1/ COL6A1/COL3A1/COL5A1

“Count” stands for the number of DE genes associated with the listed GO.ID.

**Table 5:** KEGG pathway analysis of genes with up-regulated methylation.

**Pathway ID**	**Definition**	***P*-value**	**Count**	**Genes**
rno04512	ECM-receptor interaction	2.54915E-10	83	COL1A1/COL1A2/COL4A1/COL4A2/ COL4A3/COL6A1/COL6A2/COL6A3/ COL6A6/ CD44/ FN1/HMMR, etc.
rno04151	PI3K-Akt signaling pathway	0.000162593	332	COL1A1/COL1A2/COL4A1/COL4A2/ COL4A3/COL6A1/COL6A2/COL6A3/ CCND3/CCNE1/ EFNA1/EIF4B, etc.
rno04611	Platelet activation	0.001133334	124	COL1A1/COL1A2/COL3A1/F2RL3/ FERMT3/FGG/APBB1IP/ARHGEF1/ BTK/ FGG/ITGA2/ LCP2, etc.
rno04062	Chemokine signaling pathway	0.003441997	177	NF-κB1/ARRB2/CCL9/CCR5/ CXCL12/CXCL2/CXCL9/GNB1/ GNG2/GNGT2/MAP2K1, etc.
rno00010	Glycolysis/ gluconeogenesis	0.02539777	70	GPI/HK3/LDHA/ PKM/ PCK2/ DLD/ PGAM1/ADH1/ALDH9A1/BPGM
rno04662	B cell receptor signaling pathway	0.03293257	73	NF-κB1/ BTK/CD72/DAPP1/INPP5D/ MALT1/MAP2K1 /PIK3AP1/PPP3R1/ PTPN6
rno04066	HIF-1 signaling pathway	0.0422345	108	NF-κB1/CYBB/HK3/HMOX1/ STAT3/ IFNGR1/IGF1/LDHA/MAP2K1/PFKFB3/ PRKCA/SERPINE1/TIMP1
rno04660	T cell receptor signaling pathway	0.0422345	108	NF-κB1/CBLB/CD4/CTLA4/ICOS/ LCP2/MALT1/MAP2K1/PAK1/PAK2/ PPP3R1/PTPN6/PTPRC

“Count” stands for the count of the chosen background population genes' entities associated with the listed Pathway ID.

**Table 6:** Genes associated with down-regulation of differential methylated m^6^A sites.

**Gene Name**	**Foldchange**	**FDR**	**Pathway**	**GO_MF**	**GO_BP**
ACVRL1	2.053666236	1.83832E-07	-	TGF-β activated receptor activity	TGF-β receptor signaling pathway
SMAD6	2.811467105	2.48433E-05	TGF-β signaling pathway	TGF-β receptor binding	TGF-β receptor signaling pathway
SMAD9	2.264780504	5.58215E-05	TGF-β signaling pathway	-	TGF-β receptor signaling pathway
ENG	5.169060443	1.91685E-06	-	TGF-β activated receptor activity	TGF-β receptor signaling pathway

### Expression of methylation-related enzymes in PAH lung tissue

**Figure 5:**
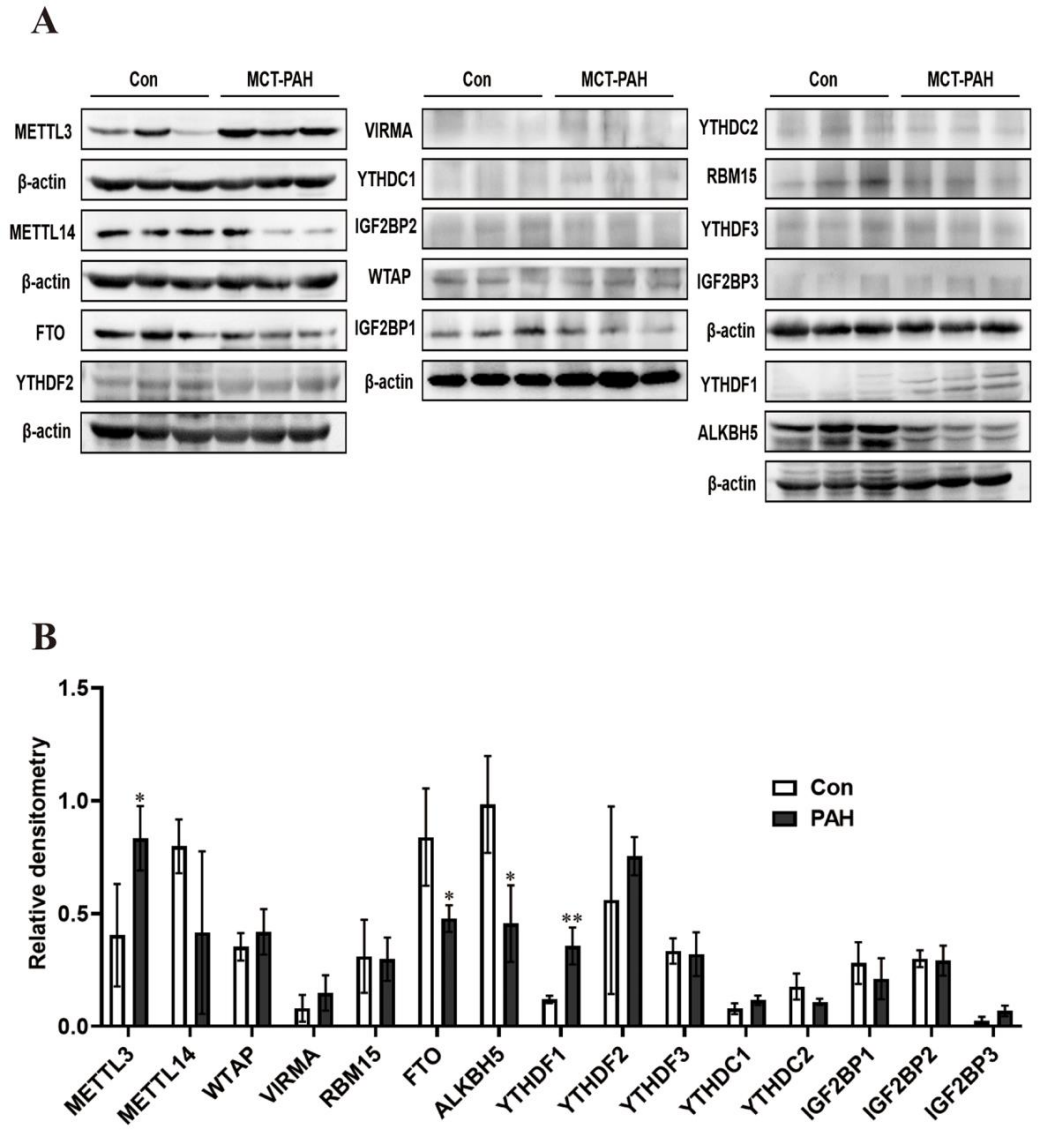
**Western blot was used to detect the expression of methylated protein in rat lung tissue.** (**A**) Western blot image of methylated modified enzyme. (**B**) Statistical graph of the expression of methylated modifying enzymes. *P < 0.05, **P < 0.01.

**Figure 6:**
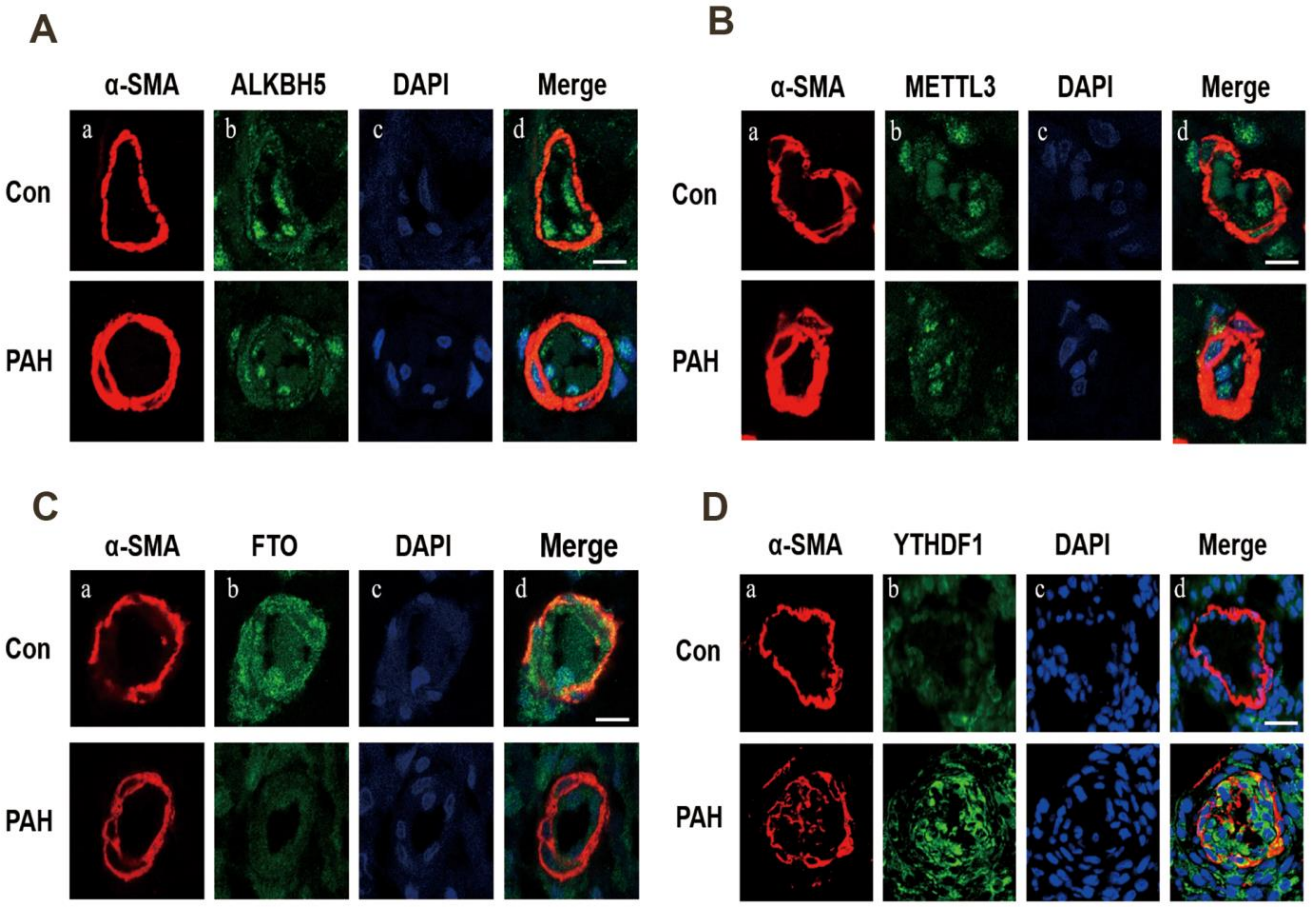
Immunofluorescence was used to detect the expression of ALKBH5 (**A**), METTL3 (**B**), FTO (**C**) and YTHDF1 (**D**) in pulmonary vessels. (**A**) Vascular staining image (red), (**B**) objective protein staining image (green), (**C**) nuclei staining image (blue), (**D**) merged images.

Western blot analysis was used to verify the expression of methylation-related enzymes in PAH. In MCT-PAH group, the expression of FTO and ALKBH5 were decreased, the expression of YTHDF1 and METTL3 were increased, while differences in the expression levels of other methylation modification-related enzymes between the two groups were not statistically significant. Then four methylation-related enzymes, that exhibited obvious changes, were evaluated via immunofluorescence. The expression of FTO, ALKBH5, METTL3 and YTHDF1 were determined by quantifying overlap between the red and green signals, which revealed a faint fluorescence intensity for FTO and strong fluorescence intensity for YTHDF1 in small pulmonary arteries of MCT-PAH rats lung tissue, while no significant differences for ALKBH5 and METTL3 were found in the pulmonary vessels (Figures 5, 6).

## DISCUSSION

m^6^A modification plays an indispensable role in the expression and critical dynamics of numerous genes [23] and has been extensively studied in various biological processes and diseases, such as multifunctional regulation and the self-renewal of embryonic stem cells [24], the growth and development of eukaryotes [25], tumors [26], and angiocardiopathy [27, 28]. In this study, we constructed a transcriptome-wide map of m^6^A-modified mRNAs in lung tissue from rats with MCT-induced PAH. We used MeRIP-seq to map the epitranscriptomic landscape of the lung tissue from PAH rats and then quantitatively compared transcriptome-wide changes between PAH and control groups. The m^6^A levels of most genes with DMMSs were increased in the lung tissue of rats with MCT-induced PAH. It is well accepted that m^6^A sites are mainly enriched in 3'UTR and near stop codons, however this study showed most of the m^6^A sites are located in CDs. The coding sequences in eukaryotes includes exons and introns. Genes containing exons can be transcribed into precursor RNA, and then the transcribed part of introns can be self-cleaved into mature mRNA, which is finally translated into proteins. m^6^A methylation is concentrated in the CDs in PAH, suggesting that the changes of signaling pathways and metabolic molecules may be closely related to the methylation changes in the coding region during the pathogenesis of PAH. These results indicate that m^6^A plays a key role in PAH.

We performed GO and KEGG analyses of coding genes harboring DMMSs, which showed that genes with increased methylation in the PAH group compared to the control group were enriched mainly in biological processes and pathways associated with the immune response. As plenty of research reported, a variety of inflammatory mediators and cytokines released by immune cells can bind the corresponding receptors on vascular endothelial cells, smooth muscle cells and fibroblasts, inducing vascular remodeling [29–32]. These results imply that m^6^A plays a significant role in the regulation of inflammation, suggesting that m^6^A modification may play an important role in PAH by regulating the inflammatory process.

In addition, the molecular functions of the up-regulated m^6^A methylation genes were related to PDGF binding and extracellular matrix structural constituents. KEGG pathway analysis demonstrated that genes with upregulated methylation participate in extracellular matrix (ECM)-receptor interaction, PI3K/AKT signaling pathway and platelet activation, which has been shown to play a major role in the pathogenesis of PAH [33–35]. Further GSEA result also showed the similar results (Supplementary Figure 1). Studies have shown that ECM remodeling occur early in the disease process, before the onset of the increase in the intimal and medial thickness and pulmonary artery pressure, suggesting that the ECM is a cause rather than a result of distal pulmonary vascular remodeling. Inhibition of ECM remodeling can prevent and reverse pulmonary arterial hypertension [33]. Interestingly, the PI3K/AKT pathway is a classical downstream signaling pathway in PAH, and its activation can promote the proliferation of pulmonary arterial smooth muscle. Previous studies by our research group confirmed that PDGF mediates the occurrence and development of PAH by activating the PI3K/AKT/mTOR/HIF-1α signaling pathway [36]. We also identified the COL1A1 (fold change=4.979) and COL1A2 (fold change=5.974) to be included in ECM-receptor interaction, PI3K/AKT signaling pathway and Platelet activation. The COL1A1 gene encodes the α1 chain of triple-helix type I collagen molecules, and type I collagen is a fibrillar collagen subtype. A few reports have shown that COL1A1 is a major cause of pulmonary artery stiffening and decreased right ventricular systolic function in hypoxia pulmonary hypertension [37–39]. GO analysis indicated that COL1A1/2 are significantly associated with PDGF binding. Considering the abovementioned observations, we hypothesize that the increased methylation of COL1A1 may activate the PI3K/AKT signaling pathway by increasing its binding to PDGF. These observations may show that m^6^A exerts an enormous effect on PDGF and the regulation of its downstream signaling pathway.

In addition, nuclear factor kappa B (NF-κB), which regulates the transcription and expression of multiple cytokines related to immunity and inflammation, exhibited significantly increased methylation in the T cell receptor signaling pathway, B cell receptor signaling pathway, chemokine signaling pathway and HIF-1 signaling pathway. Recent studies have shown that inflammation is closely related to vascular remodeling in PAH [40], suggesting that NF-κB may be involved in PAH by regulating the methylation of inflammatory factors.

Meanwhile, pathway analysis of the differential m^6^A genes also found that mRNA m^6^A levels of HK3, GPI, LDHA and PKM were up-regulated in the glycolysis/ gluconeogenesis pathway in PAH. A large number of studies have shown that m^6^A methylation can affect the proliferation and metastasis of tumor cells by regulating the process of glycolysis. A study showed that in leukemia cells, decreased FTO expression can increase the level of m^6^A methylation in mRNA of the key enzyme of glycolysis, while reading protein YTHDF2 reduces the translation and expression of LDHB, a key enzyme in glycolysis, by promoting mRNA degradation, thereby inhibiting aerobic glycolysis and cell proliferation [41, 42]. Moreover, studies have confirmed that aerobic glycolysis plays an important role in the proliferation of pulmonary artery smooth muscle cells, and inhibition of aerobic glycolysis can inhibit the proliferation and migration of pulmonary artery smooth muscle cells, and can partially reverse PAH of animal model [36]. These results suggest that m^6^A methylation may affect the protein translation or transcription of the key enzyme of glycolysis by upregulating the mRNA m^6^A level, thus participating in the development of PAH.

In addition, a small percentage of familial PAH cases are attributed to mutations in TGF-β family receptor members or related downstream signaling proteins (e.g., ACVRL1/ALK1, endoglin/ENG, and SMAD9) [43]. Studies have demonstrated that m^6^A can affect RNA stability, splicing, localization, and translation at the posttranscriptional level, thereby affecting gene expression [10]. Analysis of the MeRIP-seq results revealed that the genes harboring down-methylation m^6^A sites are primarily associated with TGF-β-activated receptor activity. More importantly, the analysis of differential methylated m^6^A sites in this study showed that multiple genes related to PAH, including ACVRL1, ENG, SMAD6 and SMAD9, had down-regulated methylation, and those genes have been found related to TGF-β signal pathway in molecular function, biological process or KEGG pathway. Previous studies have shown that TGF-β pathway plays an important role in PAH by regulating pulmonary vascular remodeling, variation of several TGF-β family receptor members, such as ACVRL1, ENG and SMAD9, are pathogenic genes of hereditary pulmonary arterial hypertension patients [6]. Combined with the above results, we speculated that m^6^A modification may participate in the development of PAH by regulating ACVRL1, ENG, SMAD6 and SMAD9.

Recent study confirmed that elevated m^6^A methylation and increased expression of YTHDF1 exist in pulmonary arterial hypertension, furthermore, YTHDF1 promotes pulmonary artery smooth muscle cells proliferation and PAH via enhancing MAGED1 translation [44]. However, there are only sporadic study concerned expression and function of methylation modifying enzymes in PAH [44, 45]. In this study, expression of methylation modifying enzyme in lung tissues of PAH rats was detected, results showed decreased expressions of FTO and ALKBH5 accompanied with increased expressions of METTL3 and YTHDF1, while there was no significant difference in expressions of other methylation modifying enzyme between PAH and control rats. Further immunofluorescence detection revealed that FTO and YTHDF1 were expressed in the small pulmonary arteries. Compared with the control group, the expression of FTO was decreased and the expression of YTHDF1 was increased in pulmonary vessels of MCT-PAH rats. FTO, a demethylase, plays an important role in DNA and RNA methylation and has been implicated in cardiac defects, including hypertrophic cardiomyopathy [46], arrhythmias [47], coronary heart disease [48] and heart failure [28]. Studies have shown that the m^6^A modification level is increased in FTO-knockdown cells and involved in various cell proliferation and migration processes [27]. As a reading protein, YTHDF1 was initially shown to bind methylated mRNA transcripts near the stop codon, which interacted with the translation initiation mechanism to improve the efficiency of the m^6^A translator and promote protein synthesis [12]. Based on the aforementioned results, we speculate changes of FTO and YTHDF1 expression may promote PAH through up-regulating the m^6^A methylation and facilitating mRNA m^6^A translation of multiple molecular function, biological process or KEGG pathway relative to PAH. These findings contribute to further understanding of the pathogenesis of PAH and provide new target for the treatment of pulmonary arterial hypertension.

## CONCLUSIONS

Our study revealed differential m^6^A methylome in the lung tissue of MCT-induced PAH rats and strong correlation between m^6^A methylation and PAH pathogenesis. Furthermore, downregulated FTO expression and upregulated YTHDF1 expression in small pulmonary arteries of MCT-induced PAH rats may play a leading role in mRNA m^6^A and involve in development of PAH through modulating inflammation, glycolysis, TGF-β family receptor members, ECM-receptor interaction and PDGF signal pathway. Thus, these findings provide a deeper understanding of epigenetics and innovative therapeutic target in PAH.

Limitations in this study still exist. First, we performed methylation sequencing analysis at the level of animal lung tissue, but further analysis of human lung tissue is needed if these findings are to be applied in the clinic. Additionally, the specific mechanism of FTO and YTHDF1 in PAH remains to be further verified.

## MATERIALS AND METHODS

### Establishment and verification of a rat model of MCT-induced PAH

This study was approved by the Institutional Animal Care and Use Committee of Hunan Children's Hospital and was conducted in compliance with the standards in the Guide for the Care and Use of Laboratory Animals. Sprague-Dawley rats (specific pathogen-free, male, 180–200 g, 6 weeks old, n=18) were obtained from Changsha Tianqin Biotechnology Company (China). The rats were randomized to the control (n=8) and PAH (n=10) group. Rats in the PAH group were intraperitoneally injected with MCT (60 mg·kg^-1^, Sigma, C2401), while rats in the control group were injected intraperitoneally with the same volume of saline [49]. All rats were housed on a 12 h light/dark cycle and given free access to food and water. After 4 weeks of feeding, the rats were anesthetized with 1% sodium pentobarbital (130 mg·kg^-1^) for echocardiography and right heart catheterization. Echocardiography was used to record the tricuspid annular plane systolic excursion (TAPSE), inner diameter of the right ventricle (RVID) and pulmonary artery blood flow acceleration time (PAAT). Images were acquired while the animals remained in the lateral decubitus position. After echocardiographic examination, the right external jugular vein was roughly dissected from the skin of the rat neck. One end of the catheter was passed through the external jugular vein into the right atrium and then into the right ventricle. The other end was connected to a pressure sensor that measured right ventricular systolic pressure (RVSP).

The rats were sacrificed by cervical dislocation after deep anesthesia. Then, heart tissues were harvested and separated. The weight ratio of the right ventricle to the left ventricle plus the ventricular septum [RV/(LV+S)] was used as an index of right ventricular hypertrophy. Lung tissues were excised and immediately frozen in liquid nitrogen or fixed in a 4% buffered paraformaldehyde solution for later RNA extraction and use in other experiments. All animal experimental protocols had been approved by the Institutional Animal Care and Use Committee of Hunan Children's Hospital.

### Histological analysis

The lung tissues obtained from each group were placed in a 4% buffered paraformaldehyde solution overnight and were then dehydrated and embedded in paraffin. Then, all lung tissues were sliced into 5 μm-thick sections, fixed on a glass slide and baked to dryness. The staining procedures were performed according to the instructions. In brief, sections were sequentially soaked in xylene, an ethanol concentration gradient and hematoxylin and then sealed with resin. After the sections had dried, the pulmonary vascular morphology was observed and imaged under an optical microscope. Finally, the pulmonary small artery wall thickness and muscularization were quantitated. Medial wall thickness (MWT) is represented as follows: MWT% = (medial wall thickness × 2/external diameter) × 100.

### RNA preparation

Four biological replicates were selected from each group, and sets of two replicates were combined into a single set. Then, total RNA was extracted from the tissues using TRIzol reagent (Invitrogen Corporation, CA, USA) in accordance with the manufacturer’s instructions. A Ribo-Zero rRNA Removal Kit (Illumina, Inc., CA, USA) was used to reduce the ribosomal RNA content in the total RNA. Then, the RNA was chemically fragmented into fragments approximately 100 nucleotides in length using fragmentation buffer (Illumina, Inc., CA, USA).

### RNA MeRIP-seq library construction and sequencing

MeRIP-seq was performed in accordance with a previously reported procedure [50] with slight modifications. In brief, MeRIP was performed with a GenSeq™ m^6^A RNA IP Kit (GenSeq Inc., China) following the manufacturer’s instructions. Both the input sample without immunoprecipitation and the m^6^A IP samples were used for RNA-seq library generation with the NEBNext® Ultra II Directional RNA Library Prep Kit (New England Biolabs, Inc., USA). The library quality was evaluated with a Bioanalyzer 2100 system (Agilent Technologies, Inc., USA). Library sequencing was performed on an Illumina HiSeq instrument with 150 bp paired-end reads.

### Western blot analysis

Protein was extracted from the rat lung tissues with lysis buffer (RIPA: PMSF=100:1), and equal amounts of protein from each sample (20 μg or 40 μg) were separated by 8% or 10% sodium dodecyl sulfate–polyacrylamide gel electrophoresis (SDS-PAGE) and transferred to polyvinylidene fluoride (PVDF) membranes. After incubation with high-affinity anti-YTHDF1 antibody (1:1000, Proteintech), anti-YTHDF2 antibody (1:5000, Proteintech), anti-METTL3 antibody (1:1000, Abcam), anti-METTL14 antibody (1:1000, Bioss Antibodies), anti-FTO antibody (1:1000, Abcam), anti-ALKBH5 antibody (1:1000, Abcam), anti-WTAP antibody (1:5000, Proteintech), anti-VIRMA antibody (1:1000, Abcam), anti-RBM15 antibody (1:1000, Proteintech), anti-YTHDF3 antibody (1:1000, Abclonal), anti-YTHDC1 antibody (1:1000, Proteintech), anti-YTHDC2 antibody (1:1000, Abclonal), anti-IGF2BP1 antibody (1:1000, Abcam), anti-IGF2BP2 antibody (1:1000, Abcam), anti-IGF2BP3 antibody (1:1000, Abcam), and anti-β-actin antibody (1:5000, Proteintech), the membranes were then incubated with peroxidase (HRP)-conjugated secondary antibody (1:5000, Proteintech). The chemiluminescent signals were detected with a chemiluminescent HRP substrate [51]. Densitometric analysis was conducted with ImageJ software.

### Immunofluorescence staining

Paraffin sections were dewaxed and placed in a repair box filled with EDTA antigen repair buffer (pH 9.0) for antigen retrieval in a microwave oven. The primary antibody was added to the slices, incubated overnight at 4° C in a wet box, and washed 3 times. After the sections had dried slightly, a fluorescence-conjugated secondary antibody raised against the species used to generate the primary antibody was added until it covered the tissue in the ring and incubated at room temperature for 50 min. DAPI was used to counterstain nuclei, and after washing, the sections were sealed with an anti-fluorescence quenching tablet. The sections were observed by laser scanning confocal microscopy, and images were acquired [52].

### Statistical analyses

In brief, paired-end reads were generated in an Illumina HiSeq 4000 sequencer and subjected to quality control by identifying bases with a quality score of Q30. Cutadapt was used to trim adaptor sequences and remove low-quality reads, after which HISAT2 was used to map clean reads to the reference genome (UCSC Rn5) [53]. Methylated sites on RNAs (m^6^A peaks) were identified by MACS (2) software [54]. Differentially methylated sites with a log2 (fold change) ≥1 and false discovery rate (FDR) ≤0.0001 were identified by diffReps [55]. The peaks identified by both software packages that overlapped with mRNA exons were determined and selected with scripts developed in-house. Gene ontology (GO) and pathway enrichment analyses of the differentially methylated protein-coding genes were performed.

GO and pathway enrichment analyses were performed with the Database for Annotation, Visualization and Integrated Discovery [56]. GO terms included three categories: cellular component (CC), molecular function (MF) and biological process (BP). The pathway enrichment analysis consisted of a functional analysis that mapped genes to Kyoto Encyclopedia of Genes and Genomes (KEGG) pathways. Fisher’s exact p-values were used to denotes the significance with which GO term or pathway enrichment correlated to the conditions. R package clusterProfiler was used for Gene Set Enrichment Analysis (GSEA), the average of all m^6^A sites fold change for each gene considered as the overall change level of the gene [57].

GraphPad Prism 8.0 software was used for analysis by single-factor analysis of variance (one-way ANOVA) for multigroup comparisons and t test for comparison between the two groups. Differences for which *P* < 0.05 were considered statistically significant. All experiments were independently repeated at least three times.

## Supplementary Material

Supplementary Figure 1
