# Supplementary figures and images for: Integrated analysis of m6A mRNA methylation in rats with monocrotaline-induced pulmonary arterial hypertension
Source: Aging (Albany NY). 2021 Jul 26;13(14):18238–56. doi: 10.18632/aging.203230 (PMC8351682; doi:10.18632/aging.203230)

SUPPLEMENTARY FIGURE

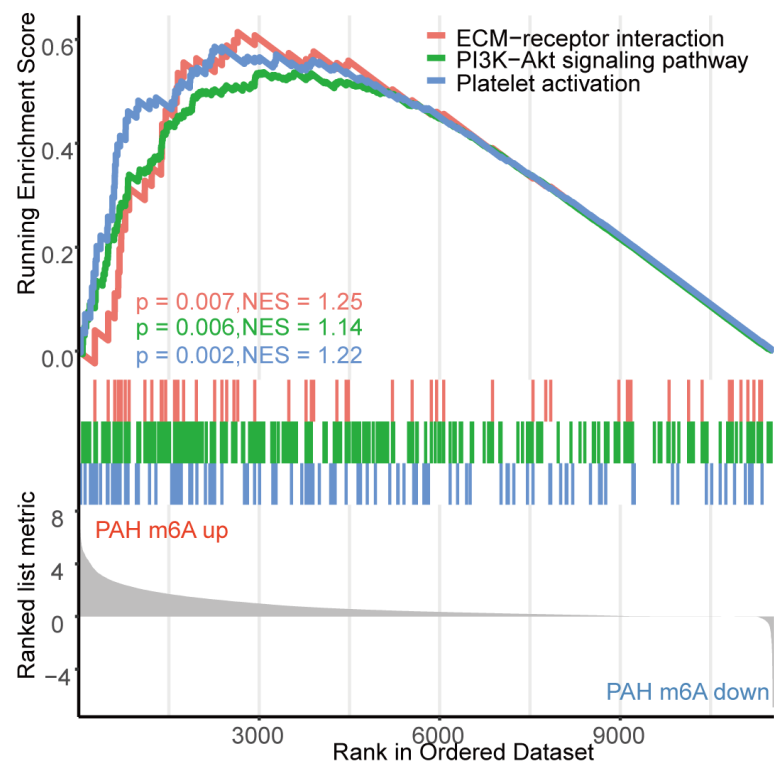

Supplementary Figure 1. GSEA analysis.

Supplement: Supplementary Figure 1 [file aging-13-203230-s001.pdf]
